# Invited Commentary: Conducting and Emulating Trials to Study Effects of Social Interventions

**DOI:** 10.1093/aje/kwac066

**Published:** 2022-04-21

**Authors:** L Paloma Rojas-Saunero, Jeremy A Labrecque, Sonja A Swanson

**Keywords:** intention-to-treat effect, per-protocol effect, randomized trials, social epidemiology, target trials

## Abstract

All else being equal, if we had 1 causal effect we wished to estimate, we would conduct a randomized trial with a protocol that mapped onto that causal question, or we would attempt to emulate that target trial with observational data. However, studying the social determinants of health often means there are not just 1 but several causal contrasts of simultaneous interest and importance, and each of these related but distinct causal questions may have varying degrees of feasibility in conducting trials. With this in mind, we discuss challenges and opportunities that arise when conducting and emulating such trials. We describe designing trials with the simultaneous goals of estimating the intention-to-treat effect, the per-protocol effect, effects of alternative protocols or joint interventions, effects within subgroups, and effects under interference, and we describe ways to make the most of all feasible randomized trials and emulated trials using observational data. Our comments are grounded in the study results of Courtin et al. (*Am J Epidemiol*. 2022;191(8):1444–1452).

## Abbreviations

ITT: intention-to-treat

***Edito*** ***r’s note:** The opinions expressed in this article are those of the authors and do not necessarily reflect the views of the* American Journal of Epidemiology.

All else being equal, if we have 1 causal effect we wish to estimate, regardless of whether it is the effect of a medication, surgery, or social policy, how should we estimate it? Ideally and when feasible, we would conduct a randomized trial with a protocol that maps onto that causal question. When a randomized trial is not feasible, we would do our best to emulate that target trial with observational data. However, studying interventions on social determinants of health often illustrates that there is not just 1 but several causal contrasts of simultaneous interest and importance, as we describe in more detail below, and each of these related but distinct causal questions may have varying degrees of available data and of feasibility in conducting trials. With this in mind, we outline some key challenges and opportunities to consider when conducting and emulating such trials, with special consideration of how to make the most of all such feasible studies. Our comments are grounded in Courtin et al.’s accompanying study (1) of the effect of randomization to a 4-fold increase in the Earned Income Tax Credit for low-income Americans without dependent children on psychological distress over 3 years of follow-up. Though our primary points apply more generally beyond social interventions, these core issues are worth underscoring because of their relevance and prevalence in answering social epidemiologic questions. Likewise, an understanding of the challenges and developments of epidemiologic methods inspired by the study of social determinants can serve as guidance for other epidemiologic areas of research.

## MAKING THE MOST OF THE RANDOMIZED TRIALS WE CAN CONDUCT

The most well-known and appreciated advantage of a randomized trial is that the participant groups randomized to each trial arm are expected to be comparable, on average, at baseline. For social epidemiology, in which sources of confounding can often be difficult to measure and adjust for, this advantage is obviously attractive (2, 3). However, randomized trials in social epidemiology are often pragmatic trials, which means that in practice they are often unblinded and vulnerable to meaningful amounts of nonadherence, competing events, and loss to follow-up (4). Each of these challenges needs to be carefully considered in light of the causal question or set of causal questions we seek to address, and we can borrow from the established literature on pragmatic trials more generally to decide how to appropriately address the challenges (4).

Consider the intention-to-treat (ITT) effect: the effect of being randomized to the social policy (e.g., randomized to be eligible for the expanded tax credit program). In settings with incomplete follow-up (such as the 69% who had outcome information at 3 years in the Courtin et al. study (1)), even estimating the ITT effect requires strong assumptions about the reasons why and how people are lost to follow-up. Selection bias due to loss to follow-up can be mitigated if we design our trials with this in mind by collecting baseline and longitudinal data on measures suspected to be related to remaining in the study. Likewise, if the outcome is potentially precluded from being observed because of a competing or truncation event, the question of interest needs to be further specified, and the trials should be designed to collect data on such events and their shared causes with the outcome (5). Because data collection itself can be a social intervention, some trials may benefit from linkage to administrative or otherwise passively collected longitudinal data to guard against participant burden—assuming, of course, that such data sets exist and contain sufficient and relevant measures.

Often stakeholders and decision-makers are also interested in the per-protocol effect (6), defined as the effect of following the intervention strategies as specified in the trial protocol. It is often inappropriately said that even with nonadherence, the ITT effect is desirable because nonadherence will also occur in the “real world,” but that logic only applies if the nonadherence in the trial perfectly mimics the degree and type of nonadherence that would occur outside the trial setting (7). Nonadherence is often related to social determinants of health, making it even more important to study nonadherence patterns and to estimate both the ITT effect and the per-protocol effect. To illustrate this, consider the trial by Banerjee et al. (8) of a multifaceted poverty reduction program including distribution of livestock, which had 52% adherence at one site in India. The reasons for nonadherence given at this site included “the (erroneous) belief that [the organization implementing the intervention] was a Christian organization trying to convert beneficiaries, and acceptance of the livestock constituted agreeing in some way to participating in Christian rituals” (8, p. 10). The ITT effect in this setting probably would not capture the effect of the intervention implemented where this particular motivation for nonadherence is absent. The degree of nonadherence also means the ITT effect is unlikely to generalize to the other sites in the study, which all had perfect adherence. This sentiment also applies to the study by Courtin et al. (1), in which the paperwork involved in receiving the tax credit within the study differed from the way the Earned Income Tax Credit is implemented outside the trial setting. In this particular case, the per-protocol effect may arguably be more transportable to other populations or contexts, depending on whether the degree and type of nonadherence would be comparable across other settings. The per-protocol effect may also more closely align in definition with some of the likely implementation strategies posttrial (since nonadherence due to the trial-specific paperwork would be a nonissue) (9). However, estimating the per-protocol effect requires strong unverifiable assumptions. Instrumental variable estimation of the per-protocol effect is one option, but the lack of blinding and other features of these trials implies that assumptions required for instrumental variable approaches may not hold (10, 11). Estimation using confounder-adjusted g-methods instead requires measuring and adjusting for reasons for and correlates of nonadherence (4, 12), and thus these assumptions may not be reasonable if nonadherence is poorly understood or the adjustment variables are not measured. Of course, the set of covariates needed for estimating the per-protocol effect may overlap with the set of covariates needed to address loss to follow-up in estimating the ITT effect, and thus the added cost (financially and in terms of participant burden) might not be substantial.

Estimating the ITT or per-protocol effect in a trial can often raise immediate questions about several adjacent treatment strategies. For example, Courtin et al. estimated the effect of randomization to eligibility for an expanded tax credit of a specified amount received annually (1), but their results raise several subsequent questions (some of which were also raised by the authors themselves): What is the effect of different amounts of tax credits at different intervals; of providing the tax credit alongside guidance or instruction for possible use; of offering the tax credit but not requiring onerous paperwork or working with a particular organization in order to obtain it? These “adjacent” questions can be viewed as novel but related interventions, new joint interventions (possibly evoking the eventual implementation elements (13)), and/or refining multiple versions of treatment into a sufficiently well-defined (or improved) intervention (14). Each of these types of “adjacent” questions can be restated as an adjacent per-protocol effect for a new target trial with new treatment strategies specified in the protocol. However, while framing the questions as new target trials is methodologically helpful, it is not meant to imply that we need to always conduct all of those other trials. In fact, our recommendation is to anticipate as many of these adjacent questions as possible whenever designing a randomized trial of a social intervention, and to collect data so that their effect estimates can be explored and estimated (with additional assumptions) by emulating the adjacent target trials within our conducted trials’ data. Ultimately, the effort to collect rich data in a trial setting can serve as an opportunity to estimate the adapted interventions of treatment strategies beyond those that defined the original trial’s protocol. Note that such data collection requires forethought and at minimum needs to include assessments of the anticipated adapted intervention definitions and associated confounders (9, 15, 16).

Understanding heterogeneity of effects across different populations is another feature evident in social epidemiology. Thus, even with perfect adherence, a single study of a social intervention in a single population is rarely broadly insightful for decision-making: A single well-conducted randomized clinical trial of a vaccine may sometimes be reasonably generalized to promoting its use to much of the world, but no single randomized trial of remote versus in-person learning could inform the questions of whether all forms of education for all forms of students in all places in the world should be performed remotely. For example, contextual variables such as quality of Internet access may be important modifiers that substantially change the magnitude, if not the direction, of the effect of trials of remote learning across different populations. Given this, trials should ideally be designed to sufficiently study relevant subgroups and allow for estimation of the effects in the target population of interest. Though estimating effects in subgroups will often require larger sample sizes, the costs of not doing so are felt inequitably when trial results get implemented (9). For example, it is easy to imagine that the “positive” results of a hypothetical randomized trial of novel remote-learning software would be utilized to support the software’s use in more schools, even though closer scrutiny of that trial might indicate that it did not include enough students with disabilities or that the effect that would have been estimated in that subgroup indicates harm.

Finally, social interventions typically imply interference. Broadly speaking, interference means that the intervention in one individual may affect other individuals’ adherence and outcomes. For example, for noncustodial parents (one of the subgroups studied by Courtin et al. (1)), the effect of receiving a tax credit may be affected by whether or not the custodial parents receive a tax credit. Interference implies there could be many questions of interest, such as direct effects of the intervention, spillover effects, and overall effects (17, 18). While there are different ways in which interference acts and affects our ability to estimate a particular effect, understanding the sources of interference and the data generation mechanisms can help prevent bias due to confounding or due to spurious associations (19). Interference also requires being clear about the level of intervention, as there are some settings in which the trial should be designed to intervene on a cluster (or network structure) level as well as or instead of at an individual level (18). When interference affects those who are eligible, researchers may consider how this interference impacts question-framing and what information on network structure is needed to address each relevant question (15, 20). Researchers might also consider effects on people who are ineligible for the intervention but networked to those who are eligible: For example, in the study by Courtin et al. (1), there is potential interest in effects on outcomes in children or custodial parents networked to the trial participants. Overall, networks highlight a key element that is often emphasized in social interventions: Context matters (and should be measured) (15, 21–23). Collecting data on the network structure is useful if not necessary, depending on the specific question being addressed.

In sum, making the most of a randomized trial—in social epidemiology and more generally—starts with embracing the idea that the ITT effect is but 1 question, and then being clear about the set of questions we seek to address with the trial data. Often, we can better answer more questions of public health relevance by collecting rich baseline and longitudinal data within the trial. When collecting such data is not feasible, bounding or bias analytical strategies may be considered (24).

## MAKING THE MOST OF ALL AVAILABLE INFORMATION

Thus far, we have considered how to calculate many effects with 1 randomized trial: the ITT effect, the per-protocol effect, adjacent per-protocol effects, effects within subgroups, and effects under interference. The spirit of how to make the most of a single randomized trial likewise applies to how to make the most of a single observational study in which we intend to emulate a trial. That is, when using observational data to emulate a target trial, we might consider doing so in ways that support estimation of many of these causal contrasts. In practice, the “adjacent” per-protocol effects described above, which can be conceptualized as distinct target trials with modifications to protocol elements, may be more easily simultaneously estimated in observational data given what happened to be measured in an available observational study compared with an available randomized trial. Coupling this insight with the risk of unmeasured confounding in the observational data setting, we have substantial reasons to use benchmarking and other triangulation strategies to bring together results from multiple study designs (21, 25). Triangulating and making the most of all available evidence ultimately requires transparency in the specific question or set of questions being asked and the underlying assumptions in any method used to produce answers. Clearly articulating the target trials’ protocol elements is a first step toward not just estimating 1 causal effect in 1 study but also identifying and seizing opportunities for bringing all of our resources together (25–27).

Finally, we acknowledge that the choice of which questions to pursue or prioritize ought to be a collaborative effort between stakeholders, community members, and researchers. Our discussion focuses on how to make the most of the available information and how to maximize the usefulness of the trials we choose to conduct. Indirectly, our discussion embraces that such collaborative efforts are likely to raise several questions and not just the ITT effect of 1 specific potential trial with 1 specific set of protocol elements. Choosing to conduct a randomized trial or to emulate a target trial with observational data to answer 1 causal question requires transparency and humility in our reliance on assumptions. The same transparency and humility are essential when trying to increase the value added by designing our trials or data collection efforts to answer multiple questions.
